# Valproic acid-induced abnormal behavior

**DOI:** 10.4103/0019-5545.58900

**Published:** 2010

**Authors:** Nanjangud Chandrashekar Nagalakshmi, Madhan Ramesh, Gurumurthy Parthasarathi, Anand Harugeri, Mary Sam Christy, Belur Seshachala Keshava

**Affiliations:** Department of Pharmacy Practice, J.S.S. College of Pharmacy, Mysore, India; 1 Department of Neurology, JSS Medical College Hospital, JSS University, Mysore, India

**Keywords:** Adverse drug reaction, behavior, valproic acid

## Abstract

A 12-year-old female was admitted to hospital with complaints of abnormal behavior. She was on valproic acid 200mg twice daily and clobazam 5mg at night for the past 13 weeks for her complex partial seizures with secondary generalized seizures. On day 60 of the treatment with valproic acid she developed behavioral disturbances and initiated treatment with tablet chlorpromazine, olanzapine and risperidone. During the present hospitalization, as there was no improvement in abnormal behavior, antipsychotics were discontinued and she was on observation for five days. On day 6, valproic acid was replaced with carbamazepine. Patient started recovering gradually from the abnormal behavior three days after the withdrawal of valproic acid and completely recovered after three months. Causality of valproic acid-induced abnormal behavior was ‘possible’. Behavioral disturbances associated with valproic acid are rare and is reversible upon discontinuation of the drug. There is a need for vigilance on abnormal behavioral effects in patients receiving valproic acid.

## INTRODUCTION

Valproic acid is commonly used in the treatment of both epilepsy and bipolar disorder. It induces transient sedation, dizziness, somnolence, reversible thrombocytopenia, and serious adverse reactions like hepatotoxicity, encephalopathy, endocrine disturbances and pancreatitis.[1] Behavioral disturbances are less frequently reported with valproic acid.[2] We report a case of valproic acid-induced abnormal behavior.

## CASE REPORT

A 12-year-old female patient weighing 35kg was admitted to hospital with complaints of behavioral disturbances since last six weeks. Her past medical history revealed that 14 weeks ago she was hospitalized following three episodes of seizures in a day with up rolling of eyeballs, confusion and limbs stiffness. A diagnosis of complex partial seizure with secondary generalized seizure disorder was made. She was treated with tablet valproic acid 200 mg twice daily and tablet clobazam 5mg at night during her hospital stay. After 5 days of treatment she was discharged with refilled prescription of valproic acid and clobazam and advised for monthly visits. Patient was otherwise normal and had no known psychiatric history or previous episodes of abnormal behavior.

After 13 weeks of treatment with valproic acid and clobazam, she was hospitalized with complaints of irrelevant talk, talking to self, wandering and singing devotional songs, decreased food intake and insomnia since about five weeks i.e. about eight weeks after initiation of valproic acid therapy. Upon hospitalization, tablet chlorpromazine 100mg once daily, tablet olanzapine 5mg thrice daily, tablet risperidone 3mg twice daily were started while she continued to receive valproic acid and clobazam at the same dose. She was discharged on day three of hospitalization with an advice to continue all medications prescribed during the hospital stay at same doses for 15 days. However, her complaints of wandering, insomnia and decreased food intake continued to persist and she was readmitted to the hospital after a week.

During the present hospital admission patient was under observation and continued to receive valproic acid and clobazam. The antipsychotics were discontinued. As there was no improvement in behavior, valproic acid was suspected as a causative agent for abnormal behavior and was replaced with tablet carbamazepine 200 mg twice daily on day six of her hospital stay. Three days after the withdrawal of valproic acid her sleep pattern, food intake and speech improved significantly. A week later she was discharged with carbamazepine and clobazam with an advice for the review after two weeks. She recovered completely after 12 weeks. Later she continued to receive only carbamazepine for her seizure disorder.

## DISCUSSION

The case we report here is, to the best of our knowledge, the first report of valproic acid- induced abnormal behavior from India. The English language search made using terms ‘valproic acid’ and ‘behavior’ or ‘wandering behavior’ or ‘verbal behavior’ or ‘speech’ or ‘personality’ or ‘REM sleep parasomnias’ in OvidSP and PubMed did not identify valproic acid induced abnormal behavior reported from India. However, the search identified six citations related to behavioral changes in patients receiving valproic acid published from other countries.

Abnormal behavior like increased talking, wandering, singing devotional songs, decreased sleep and food intake were observed in this patient following the administration of valproic acid and clobazam. These symptoms neither explained purely the bipolar affective disorder nor psychosis.[3] This patient was neither previously diagnosed to have had abnormal behavior nor had received valproic acid. The symptoms persisted for around nine weeks while she was on valproic acid and clobazam. Abnormal behavior continued to worsen despite the initiation of antipsychotic agents. This could explain the possible involvement of medications which the patient continued to receive for the treatment of seizure disorder. Of the medications received prior to the onset of abnormal behavior, clobazam is not reported to cause behavioral abnormalities.[3] Thus the valproic acid was suspected as a possible cause for the development of abnormal behavior in this case and was discontinued.

All the symptoms observed in our patient were different compared to the symptoms of valproic acid induced hyperammonemia reported in the literature.[4] Also, the patient did not receive phenobarbitone or phenytoin, which increase the risk of developing hyperammonemia to valproic acid.[5] Although, ammonia level was not measured in this case, there have been reports of developing altered mental state without hyperammonemia and elevated valproate levels.[6] Positive dechallenge with improvement in abnormal behavior in three days favors the involvement of valproic acid.[7,8] The recovery from abnormal behavior was only attributable to withdrawal of valproic acid as no specific treatment was given for the abnormal behavior after valporic acid discontinuation. Valproic acid is reported to induce behavioral disturbances following seven to 15 days of therapy.[8,9] In our case, delayed onset was observed.

Till date, the World Health Organization (WHO) international drug monitoring center has received 12 cases of abnormal behavior in patients receiving valproic acid from United States and Germany.[10] Similar reaction has been experienced by patients receiving valproic acid 1g to 2 g per day.[7,10] Comparatively, in this case, daily dose (400 mg/day) of valproic acid was low due to low body weight. Behavioral effects depend on individual drug, its efficacy and patient's biological/psychological condition.[7] The causality of reaction was ‘possible’ as assessed by WHO scale[11] and Naranjo's scale.[12] The reaction was moderate in its severity as it resulted in hospital admission.[13]

Valproic acid, carbamazepine, phenobarbital, lamotrigine, phenytoin, levetiracetam, oxcarbazepine, topiramate, zonisamide, and gabapentin produce their effects on mood and behavior in patients with epilepsy.[14] These include psychosis or affective disorders either depression or mania. Due to limited literature reports of valproic acid-induced abnormal behavior, the severity and psychopathological nature of behavioral disturbances induced by valproic acid remains obscure.[15]

The exact mechanism of valproic acid-induced abnormal behavior is not known. However, valproic acid by increasing the level of brain Gama- amino-butyric acid (GABA) provokes dose related delirium, visual hallucinations and hyperactivity, particularly in children.[16] Behavioral disturbance associated with valproic acid is rare and is reversible upon discontinuation of valproic acid. Considering similar cases being reported to WHO drug monitoring center, it is important to create awareness and alert clinicians on possibilities of valproic acid-induced abnormal behavior.
